# Legionnaires’ Disease Causing Severe Rhabdomyolysis and Acute Renal Failure: A Case Report

**DOI:** 10.5811/cpcem.2022.8.57155

**Published:** 2022-10-24

**Authors:** Andrew Branstetter, Benjamin Wyler

**Affiliations:** * Mount Sinai Morningside-West, Department of Emergency Medicine, New York, New York; † Icahn School of Medicine at Mount Sinai, Department of Emergency Medicine, New York, New York

**Keywords:** Legionella, rhabdomyolysis, renal failure, emergency, case report

## Abstract

**Introduction:**

Legionnaires’ disease is a multisystem disease involving respiratory, gastrointestinal, and neurologic systems. This is a case of a previously healthy 44-year-old man who was diagnosed with Legionella pneumonia causing acute kidney failure and rhabdomyolysis.

**Case Report:**

The patient presented with four days of chills, shortness of breath, chest discomfort, diarrhea, and myalgias. Laboratory testing revealed hyponatremia, leukocytosis, elevated inflammatory markers, renal failure, and rhabdomyolysis. He was admitted to the intensive care unit for acute hypoxemic respiratory failure, received a course of antibiotics, and more than two weeks of intermittent hemodialysis with full recovery of renal function. The pathophysiologic mechanisms by which Legionella causes rhabdomyolysis and acute kidney failure are not fully understood, although numerous mechanisms have been proposed including direct invasion of myocytes and renal tubular cells.

**Conclusion:**

Legionnaires’ disease is one of several infections that can cause rhabdomyolysis and kidney failure. Although rarely described in the literature, it is important for emergency physicians to be aware of this clinical entity in order to implement early diagnostic testing and empiric treatment.

## INTRODUCTION

We report a case of a previously healthy man who was found to have acute renal failure attributable to rhabdomyolysis in the setting of Legionella pneumonia. He quickly developed respiratory distress due to volume overload, which required treatment with high-flow nasal cannula oxygen and urgent hemodialysis. In this case report we review the patient’s initial presentation and management and highlight rare infectious causes of rhabdomyolysis of which emergency physicians should be aware.

## CASE REPORT

A 44-year-old Black man with no known medical history presented to the emergency department (ED) in March 2021 with four days of chills, shortness of breath, chest discomfort, diarrhea, and myalgias. He reported decreased urine output with darkening of his urine. He denied recent travel. He had attended a social gathering the weekend before and was motivated to come to the ED by concern that he might have acquired coronavirus disease 2019 (COVID-19). He reported occasional cannabis use. He worked in construction with a prior history of asbestos abatement work and reported complying with recommended personal protective equipment.

Initial vital signs were blood pressure 150/100 millimeters of mercury, heart rate 86 beats per minute, oxygen saturation of 98% (via 2 liters per minute nasal cannula), and a respiratory rate 20 breaths per minute. Lung exam revealed mildly decreased breath sounds at the bilateral bases without wheezes or rales. No lower extremity edema was noted. Within a few hours of arrival, the patient subsequently became tachypneic to greater than 40 breaths per minute with increased work of breathing. Point-of-care ultrasound was performed and revealed bilateral B-lines, more prominent in the left lung.

**Image 1:**
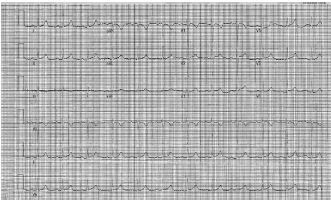
Electrocardiogram revealing normal sinus rhythm, heart rate 72 beats per minute, corrected QT interval 477 milliseconds.

**Image 2:**
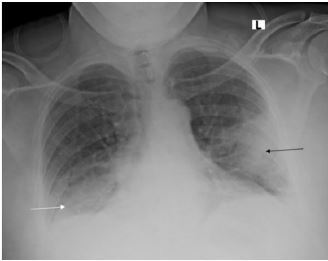
Chest radiograph with bilateral infiltrates involving the left mid to lower lung (black arrow) and the right lung base (white arrow), suggestive of multifocal or viral pneumonia.

Electrocardiogram showed normal sinus rhythm without signs of ischemia or infarction (Image 1). Chest radiograph demonstrated bilateral infiltrates suggestive of multifocal or viral pneumonia (Image 2). Serum chemistries and complete blood count measured blood urea nitrogen (BUN) 123 milligrams per deciliter (mg/dL) (reference range: 7–20 mg/dL); creatinine (Cr)14.6 mg/dL (0.7–1.3 mg/dL); sodium 121 millimoles per liter (mmol/L) (135–145 mmol/L); anion gap 25 mmol/L (7–16 mmol/L); glucose 289 mg/dL (60–100 mg/dL); creatine phosphokinase 36,716 units per liter (U/L) (30–200 U/L); and a white blood cell count 30.6 × 10^3^ leukocytes per microliter (K/uL) (4.5–11.0 K/uL) with 82% neutrophils (40.0–72.0%).

Other diagnostic tests, including lactate dehydrogenase (LDH), erythrocyte sedimentation rate, C-reactive protein (CRP) and procalcitonin measured 1,010 U/L (100–220 U/L); 114 millimeters per hour (mm/hr) (0–13 mm/hr), 476 milligrams per liter (mg/L) (<5.1 mg/L), and 32.58 nanograms per milliliter (ng/mL) (no reference range provided), respectively. Two sequential reverse transcription polymerase chain reaction severe acute respiratory syndrome coronavirus-2 tests were negative. A Foley catheter was placed with only 50 mL of dark urine output collected in two hours. Urinalysis revealed amber colored, cloudy urine, with protein greater than 500 mg/dL (reference range: negative mg/dL); ketones 150 mg/dL (negative mg/dL); large blood (reference range: negative); and 4 red blood cells (RBC) per high-power field (HPF) (0–3 RBC/HPF).

The patient was administered both high-flow nasal cannula oxygen and empiric broad spectrum intravenous (IV) vancomycin, ceftriaxone, and azithromycin, for presumed bacterial pneumonia. The patient was then admitted to the medical intensive care unit for hypoxemic respiratory failure attributed to the combination of pneumonia and volume overload in the setting of renal failure.

**CPC-EM Capsule**

What do we already know about this clinical entity?

*Legionnaires’ disease involves respiratory, gastrointestinal, and neurologic systems. Rarely, this disease can cause acute renal failure and severe rhabdomyolysis*.

What makes this presentation of disease reportable?

*This is a rare presentation of a common disease. Previous cases of rhabdomyolysis in Legionnaires’ disease involved older patients with multiple comorbidities*.

What is the major learning point?

*Emergency physicians should include Legionnaires’ disease in the differential for patients with pneumonia and acute kidney dysfunction*.

How might this improve emergency medicine practice?

*By testing more frequently for Legionnaires’ disease and initiating antibiotics early in the patient emergency room visit, emergency physicians can improve patient outcomes*.

Subsequent testing on hospital day one showed a positive Legionella urine antigen. He was treated with IV levofloxacin until hospital day 3 but required azithromycin due to a skin rash. He completed a two-week course of antibiotic therapy. He received intermittent hemodialysis for 16 days while in the hospital and was discharged home on hospital day 23 with improvement in renal function (BUN 22 mg/dL, Cr 1.88 mg/dL). At follow-up nephrology visit approximately one and one-half months later, the patient had no residual symptoms and showed further improved kidney function (BUN 15 mg/dL, Cr 1.14 mg/dL).

## DISCUSSION

Legionella is a genus of Gram-negative, aerobic organisms known to cause disease in humans. Legionellosis is classically transmitted through stagnant water, such as cooling towers for air conditioning and pipes for water distribution in large buildings; thus, infection is more prevalent in urban areas than in rural areas.^1^,^2^ While this patient had a distant history of construction work, there was no known occupational link to his Legionella infection.

*Legionella pneumophila*, the most studied species, can cause a spectrum of human diseases that ranges from Legionnaires’ disease—a severe multisystem disease with pneumonia, gastrointestinal, musculoskeletal, and neurologic involvement—to Pontiac fever, a self-limited array of mild flu-like symptoms.^1^ Because Legionnaires’ disease can present with a wide array of signs and symptoms, clinicians have attempted to risk-stratify patients’ symptoms to determine the likelihood of infection being due to Legionella. Six clinical factors serve as a clinical prediction tool including elevated body temperature; absence of sputum; low serum sodium; high levels of LDH and CRP; and low platelet counts. This tool conferred a negative predictive value of 99.4%, which reliably rules out Legionella infection when fewer than two features are present.^3^

The Infectious Diseases Society of America and the American Thoracic Society (IDSA/ATS) guidelines recommend Legionella antigen testing for any patient with one of five risk factors: ICU admission; failure of outpatient antibiotics; active alcohol misuse; travel within the prior two weeks; or pleural effusion.^4^ However, a 2011 retrospective study found that as many as 41% of Legionella cases would have been missed by application of the IDSA/ATS guidelines alone, which suggests that more liberal Legionella testing may be necessary to avoid adverse clinical outcomes.^5^ The COVID-19 pandemic has further confounded diagnosis of atypical pneumonias such as Legionella, as many clinicians may focus on the diagnosis of COVID-19 instead of others.^6^

The first case of rhabdomyolysis attributed to Legionnaires’ disease was reported in 1980.^7^ Many questions remain regarding the pathogenic mechanisms behind rhabdomyolysis and renal failure in Legionnaires’ disease. Proposed mechanisms include toxin generation leading to microvascular vasoconstriction and muscle ischemia, and direct bacterial invasion of myocytes, as supported by calf muscle biopsies in a 1991 study.^8^,^9^ Legionella can also cause acute kidney injury in the absence of respiratory involvement, in contrast to many other pneumonic bacteria.^10^ Muscle damage with ensuing kidney injury can increase mortality of Legionnaires’ disease by up to 40%, necessitating the early recognition and initiation of IV antibiotics, IV resuscitation, and possible hemodialysis.^8^ In addition, Legionella infection is believed to cause kidney injury by other mechanisms independent of rhabdomyolysis. A 2000 review of kidney biopsies in Legionella patients found that cytopathology ranged from tubulointerstitial nephritis (TIN) to acute tubular necrosis (ATN) and glomerulonephritis.^11^ For this reason, steroids may be beneficial to recovery of renal function in the setting of TIN. 8,11 Based on initial urine studies, this patient had a presumed TIN or ATN etiology due to his poor renal function.

Legionella is one of several infectious etiologies of rhabdomyolysis. A 2020 systemic review reported that among organisms causing atypical pneumonia and myositis, the majority were Legionella, with rarer causes including *Mycoplasma pneumoniae*, *Francisella tularensis, Coxiella burnetii*, and *Chlamydia psittaci*.^12^ Many viruses are also associated with rhabdomyolysis, most commonly influenza, human immunodeficiency virus, and Coxsackie viruses.^8^,^13^

To our knowledge, there has been only one previous report of rhabdomyolysis and renal failure complicating Legionella infection discussed in the emergency medicine literature; this involved a patient with several medical comorbidities and also a subacute presentation of signs and symptoms over two to three weeks.^14^ In contrast, the patient described in this case report developed an acute presentation, lacked comorbidities, and required urgent hemodialysis within 24 hours of ED presentation.

## CONCLUSION

Legionnaires’ disease is a rare cause of rhabdomyolysis and renal failure. Case reports have generally described patients with multiple comorbidities unlike the patient described above. Despite a US Centers for Disease Control and Prevention surveillance of all Legionella cases, there is no published data on the incidence of these complications among these patients. Emergency physicians should be cognizant both that Legionella can develop in those without co-morbid conditions and, further, the disease can cause rhabdomyolysis and renal failure. Emergency physicians should have increased suspicion for Legionella in patients with hyponatremia, elevated inflammatory markers, pleural effusions, and a history of alcohol misuse.
